# Plant quercetin degradation by gut bacterium *Raoultella terrigena* of ghost moth *Thitarodes xiaojinensis*

**DOI:** 10.3389/fmicb.2022.1079550

**Published:** 2022-12-22

**Authors:** Guiqing Liu, Li Cao, Richou Han

**Affiliations:** Guangdong Key Laboratory of Animal Conservation and Resource Utilization, Guangdong Public Laboratory of Wild Animal Conservation and Utilization, Institute of Zoology, Guangdong Academy of Sciences, Guangzhou, Guangdong, China

**Keywords:** *Spodoptera litura*, *Thitarodes xiaojinensis*, *Raoultella terrigena*, herbivory, plant secondary metabolites, adaptation, microbiota, detoxification

## Abstract

Associated microbes of several herbivorous insects can improve insect fitness. However, the contribution of specific insect gut bacterium to plant toxin toxification for its host fitness remains scarce. Here, a gut bacterium *Raoultella terrigena* from the ghost moth *Thitarodes xiaojinensis* larvae was identified. This bacterium grew unhindered in the presence of *Polygonum viviparum*, which is a natural food for ghost moth larvae but showed significant growth inhibition and toxicity against *Spodoptera litura*. *S. litura* reared on artificial diets containing 5, 15 and 25% *P. viviparum* powder after 7 days coculture with *R. terrigena* were found to have shorter larval and pupal durations than on the diets containing *P. viviparum* powder but without *R. terrigena* coculture. HPLC analysis revealed that the content of quercetin in mineral medium containing 15% *P. viviparum* powder after 7 days coculture with *R. terrigena* was significantly decreased (79.48%) as compared with that in *P. viviparum* powder without *R. terrigena* coculture. *In vitro* fermentation further verified that *R. terrigena* could degrade 85.56% quercetin in Lucia-Bertani medium. *S. litura* reared on artificial diets containing 0.01, 0.05 and 0.1 mg/g quercetin after 48 h coculture with *R. terrigena* were also found to have shorter larval, prepupal and pupal durations, as well as higher average pupal weight and adult emergence rate than on the diets containing quercetin, but without *R. terrigena* coculture. In addition, *R. terrigena* was detected in the bud and root tissues of the sterilized *P. viviparum*, indicating that *T. xiaojinensis* larvae might acquire this bacterium through feeding. These results demonstrate that the gut bacteria contribute to the degradation of plant toxic molecules to improve the development of herbivorous insects and provide fundamental knowledge for developing effective methods for beneficial insect rearing and pest control.

## Introduction

The interaction between insect and plant is often mediated by plant secondary metabolites. These molecules not only act as feeding attractants, but also as feeding deterrents or toxins to herbivorous insects by impeding digestion, hindering normal metabolism, or blocking nutrient transport, etc., (Mithöefer and Boland, 2012). In order to use plants as food sources, insect herbivores have to equip weapons to overcome a variety of plant defenses, including detoxifying plant secondary compounds. An array of xenobiotic detoxification enzymes such as P450, carboxylesterase and glutathione S-transferases in insects play an indispensable role in antioxidant defense and detoxification of plant secondary metabolites and exogenous xenobiotics (Li et al., 2007; Zhang et al., 2013). Recent evidence suggest that their microbiome vary across life stages and may affect a wide variety of host traits including fitness and immunity (Beran and Gershenzon, 2016; Ali et al., 2018; Itoh et al., 2018; Ali et al., 2019; Zaynab et al., 2019; Khan et al., 2020). Furthermore, insect gut is the main site of action of toxic plant metabolites and detoxification reaction. To help counter toxic effects of these defense compounds, several insects such as generalist lepidopteran herbivores cotton leafworm *Spodoptera littoralis*, coffee berry borer *Hypothenemus hampei*, pine weevil *Hylobius abietis*, leaf-cutter ant *Atta colombica* and *A. cephalotes* have been found to harbor gut microbiota with the metabolic ability to degrade plant secondary metabolites (Schramm et al., 2012; Ceja-Navarro et al., 2015; Berasategui et al., 2017; Francoeur et al., 2020).

The traditional medicinal plant *Polygonum viviparum*, belonging to Polygonaceae family, is a perennial herb and widely distributed in the alpine meadows at an altitude of 2,220–5,100 (Li and Shen, 1996). Most *Polygonum* plants have insecticidal, antifeedant and repellent activities, among which *P. hydropiper* has long been applied as plant insecticide and antifeedant (Zeng et al., 2008). It is deemed to be an effective biocide against many insect pests such as aphid *Myzus persicae*, armyworm *Mythimna separata*, diamond back moth *Plutella xylostella*, cabbage caterpillar *Pieris rapae* and rice planthopper *Nilaparvata lugens*, as well as confused flour beetle *Tribolium confusum* (Zeng et al., 2008). The acetone extracts from the whole plant of *P. viviparum* showed certain insecticidal activity against *M. seperata* (Li et al., 2009). The root of *P. viviparum* also showed significant growth inhibition and toxicity against the two important agricultural pests *P. xylostella* and *Spodoptera litura* (Qiu and Han, 2016). However, which secondary metabolites from *P. viviparum* inhibit insect growth remains unknown.

Major secondary metabolites of *P. viviparum* are flavonoids, include groups of bioactive gallic acid, quercetin and chlorogenic acid (Zhang et al., 2005; Li et al., 2014). Flavonoids are one of the largest and most diverse groups of plant secondary metabolites, with more than 6,500 diverse flavonoids being identified in plants (Ververidis et al., 2007). These compounds have been found to negatively affect development and behavior of insect herbivores from Hemiptera, Coleoptera, Lepidoptera, and Hymenoptera, by acting as feeding deterrents, growth inhibitors or toxins (Elliger et al., 1981; Lattanzio et al., 2000; Nyman and Julkunen-Tiitto, 2000; Salunke et al., 2005; Schnarr et al., 2022). Thus, these metabolites might be involved in *P. viviparum* resistance to herbivores.

However, the root of *P. viviparum* is the main food for the ghost moth larvae (Lepidoptera: Hepialidae: *Thitarodes*), an obligate host of the medicinal fungus *Ophiocordyceps sinensis* in the Tibetan plateau. The Chinese cordyceps formed by this parasitism has been considered as one of the most valued health food and traditional Asian medicine since the 15th century (Sung et al., 2007; Zhou et al., 2014; Han et al., 2019). Previous studies have demonstrated the flavonoids degradation by some bacterial species such as *Bacteroides fragilis*, *B. uniformis*, *Bifidobacterium pseudocatenulatum*, *Clostridium perfringens*, *Enterococcus avium* and *Flavonifractor plautii* in the human gut (Zhang et al., 2014; Braune and Blaut, 2016). However, whether flavonoids of *P. viviparum* exhibit inhibitory effect against *S. litura*, and whether gut microorganisms from the ghost moth larvae are involved in the detoxificationof the compounds in *P. polygonum* is still unknown.

In this study, *R. terrigena* was isolated from *P. viviparum* and the gut of *Thitarodes xiaojinensis* larvae which consumed the root powder of *P. viviparum* as a medium component. This bacterium significantly decreased the content of quercetin in *P. viviparum* root powder and was further demonstrated to degrade 85.56% quercetin by *in vitro* fermentation. This bacterium also improved the growth of *S. litura* which fed on the artificial medium containing the root powder of *P. viviparum* or quercetin. These results suggested that gut bacteria of ghost moth *T. xiaojinensis* larvae would involve in detoxifying *P. viviparum* flavonoids and thereby contributing to the adaptation of herbivorous insect to host plant. This study will not only help to understand the coevolutionary relationship between the insect and plant, but also provide new insights for artificial rearing of beneficial insects and for pest control.

## Materials and methods

### Insect and plant

*Thitarodes xiaojinensis* larvae, seeds and roots of *P. viviparum* were obtained from the known distribution areas of the Chinese cordyceps in the Xiaojin County, Sichuan Province, China (Liu et al., 2021). The larvae were collected alive and placed one larva in each vial filled with moss. All the larvae and roots were sent to the laboratory in foam boxes with ice packs wrapped with cotton to maintain the low temperature immediately after the collection. The collected larvae were identified by the amplification of the mitochondrial cytochrome b gene (cytb) sequence with the primers CB1 (TATGTACTACCATGAGGACAAATATC) and CB2 (ATTACACCTCCTAATTTATTAGGAAT) (Zou et al., 2010). *S. litura* larvae were artificially reared on a diet containing 15% wheatgerm powder, 4% yeast powder, 0.5% sucrose, 0.4% vitamin C, 14% agar, 0.4% sorbic acid and 0.4% methylparaben at 25°C, 70% relative humidity and a 12: 12 h L: D photoperiod.

### Characterization of the bacteria from *Thitarodes xiaojinensis* larval gut and *Polygonum viviparum*

Bacteria were isolated from the gut of *T. xiaojinensis* larvae and screened for the utilization of *P. viviparum* roots. Twenty *T. xiaojinensis* larvae were dissected and gut contents were macerated in 200 μl of 1 × phosphate buffer (pH = 7.4) and further diluted at a proportion of 1: 10 with 1 × phosphate buffer. Fifty microliters of the diluted gut suspension were spread on agar plates with mineral medium (9.5 mM KH_2_PO_4_, 4.8 mM MgSO_4_, 0.1 mM CaCl_2_, 0.8 mM Na_2_HPO_4_ and 10 g/l bacto agar), and a gradient of *P. viviparum* root powder ranging from 10 to 25% in weight. The *P. viviparum* root powder was prepared by a blender after the roots were washed by distilled water and dried at 25°C for 1 h. The agar plates were incubated in the dark at 13°C and 25°C, respectively. The growth of bacteria was examined every day and the morphologically distinct colonies were picked and further purified by quadrant streaking on LB (Lucia-Bertani) agar plates. Purified colonies were transferred to liquid mineral media containing 25% *P. viviparum* powder for confirming their growth. Each treatment was repeated three times.

The culture-dependent endophytes of *P. viviparum* were isolated for the first time by using the blendor method. Seeds of *P. viviparum* were surface sterilized using 10% NaClO for 30 min following by 10% formaldehyde for 20 min, and then washed using sterilized water for 5 times. Sterilized seeds were cultivated on mineral media at 15°C. Then, bud and root tissues from the sterilized seeds were used to isolate endophytic microbes. Prior to mortar and pestle maceration, unsterilized and surface-sterilized seeds were placed on LB medium to determine the effectiveness of the sterilization. An abundance of bacterial and fungal colonies was observed on the medium with unsterilized seeds, but no colony was observed on the medium plates with surface-sterilized seeds (Supplementary Figure S1). The bud and root tissues from surface sterilized seeds were then macerated and used as inoculum on plates containing modified Jensen’s medium (Gushgari-Doyle et al., 2021) and LB medium. Distinct colonies were purified by streaking on LB plates and identified as described above.

Bacterial isolates which grew on both agar plates and liquid fermentation of mineral medium containing *P. viviparum* powder were further characterized. For physiological and biochemical characterization, the bacteria were cultured on LB agar plates at 37°C for 24 h in the dark. Gram staining, casein hydrolysis, catalase, citrate utilization, gelatin liquefaction, hydrogen sulfide, indole, methyl red, nitrate reduction, oxidation and fermentation of glucose, phenylalanine deaminase, starch hydrolysis, urease and Voges-Proskauer tests were performed using standardized procedures of Bergey’s Manual of Systematic Bacteriology (Buchanan and Gibbons, 1984; Dong and Cai, 2001). For molecular characterization, DNA was extracted from bacterial cultures grown at 37°C for 24 h using an Ezup Column Bacteria Genomic DNA Purification kit (Sangon Biotech, Shanghai, China) according to the instructions. The prokaryotic V4 region of 16S rRNA was amplified with primers 27F (5’-AGAGTTTGATCCTGGCTCAG-3′) and 1492R (5’-TACGGYTACCTTGTTACGACTT-3′). The PCR product was separated by electrophoresis and the band was cut from the gels for purification, and then sequenced in Sangon Biotech (Shanghai) Co., Ltd., (Guangzhou, China). Sequence of the isolated bacterium was compared with non-redundant GenBank library using nucleotide blast search.^1^ The taxonomically related sequences were collected from NCBI Taxonomy Homepage. Phylogenetic tree was constructed by using Neighbor-Joining analysis in MEGA 5.10 software. The confidence at each node was assessed by 1,000 bootstrap replicates.

### Effect of *Raoultella terrigena* on the development of *Spodoptera litura* feeding on *Polygonum viviparum*

A gut bacterial isolate identified as *R. terrigena* was found to grow well in mineral medium containing 25% *P. viviparum* powder. Thus, this bacterial species was used for further experiments. Since no proper artificial media have been formulated for *T. xiaojinensis* larvae, a worldwide vegetable pest *S. litura* which can be reared on artificial media was used instead of *T. xiaojinensis* to determine the effect of *R. terrigena*. One milliliter of bacterial suspension with an optical density of 0.8 at 600 nm was inoculated in a 250 ml flask containing 100 ml mineral media supplemented with 20% *P. viviparum* powder. The flasks were then incubated for 7 days in a shaker at 200 rpm at 37°C. The fermented broths were divided into two parts: one part contained living *R. terrigena* bacteria and another part was autoclaved at 121°C for 10 min. The autoclaved and unautoclaved bacterial cultures were centrifuged at 2000 *g* for 5 min at 25°C. The precipitates from autoclaved and unautoclaved bacterial cultures were directly mixed with the artificial diet of *S. litura* according to the proportions of 5, 15 and 25%, respectively. The autoclaved and unautoclaved *P. viviparum* deposits without *R. terrigena* inoculation were mixed with the artificial diet in the above ratios and used as the control diets.

Twenty newly molted fourth instar larvae of *S. litura* were shifted to a sterile plastic container (diameter = 9 cm) padded with two sheets of sterile filter paper (Whatman no. 3). The larvae were fed on the diets containing 0, 5, 15 and 25% of the autoclaved and unautoclaved *P. viviparum* deposits, respectively. The diets were replaced every 24 h. Five replicates were established for each diet with 20 larvae for each replicate. The mortality of larvae and pupae, and the developmental duration in each larval and pupal stage were recorded. All the pupae were weighed on the first day post pupation.

### HPLC analysis of quercetin and gallic acid in *Polygonum viviparum* cocultured with *Raoultella terrigena*

To investigate whether the growth improvement of *S. litura* fed on the diets containing *R. terrigena*-fermented *P. viviparum* root powder was caused by the reduction of flavonoids content, the contents of quercetin and gallic acid in *P. viviparum* precipitates in the cultures with and without *R. terrigena* inoculation post 7 days at a 200 rpm shaker at 37°C were analyzed by high performance liquid chromatography (HPLC). The precipitates prepared by centrifugation as described above were freeze-dried by using Savant Modulyo D freeze drier (Thermo Fisher Scientific, Waltham, MA, United States). For each treatment, 0.5 g of dry sample was diluted to 50 ml with 30% methanol, and left for 2 h before ultrasonic extraction for 30 min and then used for HPLC analysis with a photodiode array Detector (1,100 series equipment, Agilent Technologies, Santa Clara, CA, United States). A C18 reversed-phase column (3.0 by 150 mm, 2.7 um) was used with the following parameters: 370 nm, 0.4 ml/min flow rate, and 10 μl injection volume for quercetin analysis, and 272 nm, 0.25 ml/min flow rate, and 1 μl injection volume for gallic acid analysis. The mobile phase consisted of 0.1% acetic acid in water and methanol. The peaks of standard quercetin (Macklin, Shanghai, China) and gallic acid (Macklin, Shanghai, China) under the above conditions were identified (Supplementary Figure S2).

### Proof and quantitative test of quercetin degradation by *Raoultella terrigena* bacteria

Quercetin degradation by *R. terrigena* was further verified with the following procedures. *R. terrigena* was inoculated into LB medium at a 200 rpm shaker at 37°C for 16 h. Then 10 μl of *R. terrigena* culture with an optical density of 0.8 at 600 nm was inoculated into a vial of 990 μl LB medium supplemented with and without 0.004% quercetin. Ten microliters LB medium was inoculated into the control vial with 0.004% quercetin as a control. The vials were shaken at 200 rpm at 37°C for 48 h, and the resulting cultures were centrifuged at 5000 rpm for 5 min at 25°C to pellet bacterial cells and the supernatants were taken for quercetin analysis according to the method (Juan and Chou, 2010; Zhang et al., 2014). Briefly, 1 ml supernatant was mixed with 2.5 ml methanol. The centrifuged methanol extraction was then mixed with the same volume of 2% aluminium trichloride (AlCl_3_; Macklin, Shanghai, China) in methanol. The absorbance of supernatant was measured under 428 nm with UV Spectrophotometer (Unico UV-2800A, Shanghai, China). The degradation rate of quercetin by *R. terrigena* was determined using a standard curve with quercetin (Macklin, Shanghai, China; 0–0.05 mg/ml) as the standard. All tests were replicated three times.

Both bacterial and human pirins exhibit quercetinase activity, which can be inhibited by the addition of inhibitors of the quercetin 2,3-dioxygenase reaction (Adams and Jia, 2005). To investigate the transcription of the two genes in *T. xiaojinensis*, *yhhw1* coding a pirin protein and *yhhw3* coding a quercetin 2,3-dioxygenase were firstly cloned from *R. terrigena* and RT-PCR was performed on different templates including total RNA, cDNA and DNA. Total RNA and DNA were co-extracted and separated from whole *T. xiaojinensis* larvae collected from the natural location by TRIzol reagent (Invitrogen, Carlsbad, United States) and 1 μg RNA was used to synthesize the first strand cDNA in 20 μl-reaction system following the manufacturer’s instruction for the TransScript™ One-Step gDNA Removal and cDNA Synthesis SuperMix (TransGen, Beijing, China). The primers used for RT-PCR amplifications were TGGCTCGATTCCTGGCATAC and TGACGACCTGAATCCACACG (*yhhw1*), TGCCGGCCAAAGACAAAATG and AAATGGGCCATAGCCGACAA (*yhhw3*).

### Effect of quercetin on the development of *Spodoptera litura*

To assess the effect of quercetin on the development of *S. litura*, the contact and feeding toxicities of quercetin against the newly molted fourth instar larvae of *S. litura* were investigated. For the contact toxicity assay, quercetin at three different concentrations (1, 5 and 10 mg/ml) in 1 ml methanol was, respectively, sprayed onto a sterile filter paper (Whatman no. 3: 9 cm diameter). One milliliter methanol was used as a control. After being dried under a fume hood for 10 min at 25°C, two filter papers were placed on the bottom of a sterile plastic box (14.6 × 8.5 × 5.0 cm). Fifteen fourth instar larvae of *S. litura* were introduced on the plastic box and fed with the artificial diet after 24 h. Each treatment was replicated six times. The treated larvae were maintained at 25°C in the dark. The diets were renewed and the larval mortality was observed every 24 h.

For the feeding toxicity assay, the fourth instar *S. litura* larvae were fed with 11 different types of diets containing three concentrations of quercetin without *R. terrigena*, and three concentrations of quercetin with living or dead (autoclaved) *R. terrigena*. 0.05% dimethyl sulfoxide (DMSO) as a solvent and LB medium without bacteria and quercetin were used as controls. Fifty microliters 5 mg/ml, 25 mg/ml and 50 mg/ml quercetin were added to 940 μl LB medium, respectively, and 10 μl *R. terrigena* culture (OD_600_ = 0.6) or 10 μl LB medium were added to give quercetin concentrations of 0.25, 1.25 and 2.50 mg/ml, respectively. Inhibition zone experiment had shown that quercetin at 0.25, 1.25 and 2.50 mg/ml had no inhibitory effect on the growth of *R. terrigena* (Supplementary Figure S3). After shaken at 200 rpm and 37°C for 48 h, 4 ml bacterial culture containing different concentrations of quercetin were transferred, respectively, to 96 g artificial diets which were homogenously mixed and ready to feed on the insect larvae. Each treatment was replicated five times. The treated larvae were maintained at 25°C and a 12: 12 h L: D photoperiod. The diets which were uneaten were removed and replaced with fresh diet every 24 h. The developmental duration of each larval and pupal stage was recorded. All the pupae were weighed on the first day post pupation.

### Data analysis

Results were presented as mean values with their standard deviation (SD). PROC Nonparametric Tests with the command ‘Sample K-S’ were used for the analysis of the normal distribution of data (SPSS17.0, SPSS Inc., Chicago, IL, United States). To determine if variables differed among treatments, data were analyzed by one-way ANOVA, using Tukey’s honestly significant difference test (*p* < 0.05) or *t*-tests for experiments with two treatments. GraphPad Prism 7.04 (GraphPad Software, Inc., United States) was used for creating scientific graphs.

## Results

### Gut bacteria from *Polygonum viviparum*-based medium and endophytes from *Polygonum viviparum* seeds and roots

**Figure 1:**
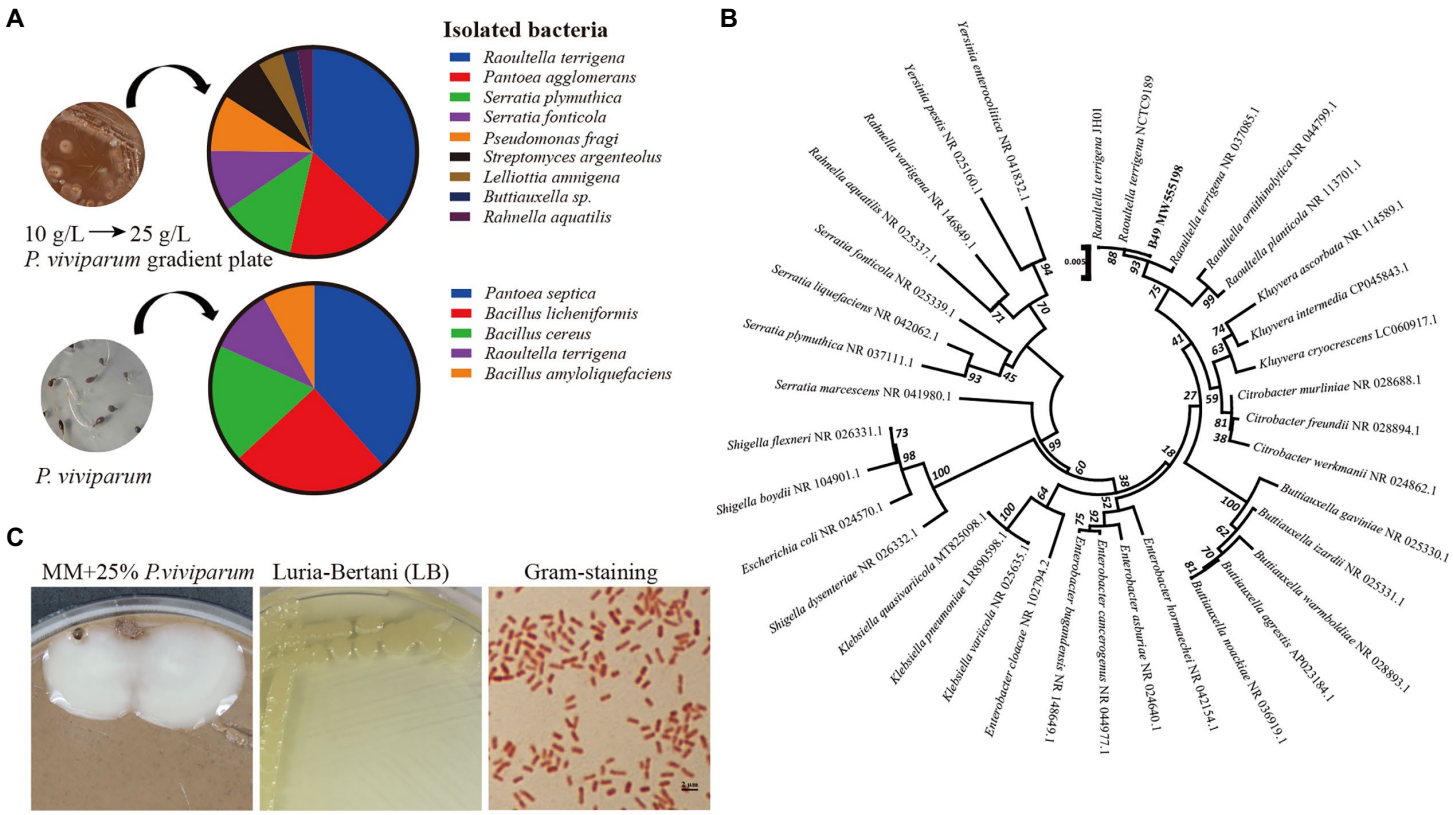
Characterization of the bacteria subsisting on *Polygonum viviparum*. **(A)** Identified bacterial species from the gut of *Thitarodes xiaojinensis* larvae on the mineral medium (MM) containing 10% *P. viviparum* root powder and from surface sterilized *P. viviparum* roots or seeds on LB medium (Lucia-Bertani) agar plates, **(B)** Phylogenetic tree of *R. terrigena* based on 16S rRNA by using the Neighbor-Joining method and 1,000 bootstrap replicates in MEGA 5.0 software, **(C)** Morphology of *R. terrigena* on MM containing 25% *P. viviparum* and LB after 24 h of culture, and a gram stain (×100).

More than 300 bacterial colonies including 9 bacterial species from the larval gut of *T. xiaojinensis* grew on the mineral agar medium containing 10% *P. viviparum* powder (Figure 1A), and 4 species including *Raoultella terrigena* (MW555198), *Pantoea agglomerans* (MW555193), *Serratia plymuthica* (MW555201) and *Rahnella aquatilis* (MW555197) on the mineral agar medium containing 25% *P. viviparum* powder. However, only *R. terrigena* normally proliferated in liquid mineral medium containing 25% *P. viviparum* powder.

Dozens of colonies comprising 5 bacterial species were obtained from bud and root tissues of the surface sterilized *P. viviparum* seeds. *R. terrigena* was also detected in bud and root tissues of *P. viviparum* (Figure 1A). The isolated *R. terrigena* strain B49 shared 100% identity to *R. terrigena* strains JH01 and NCTC9189, and clustered into a clade with other species of *Raoultella* (Figure 1B).

The isolated *R. terrigena* was cultured on mineral medium containing 25% *P. viviparum* and LB medium at 37°C for 24 h. The colonies were regular round, opaque and convex. The bacterial cells were rod-shaped, single or short chain arranged. The cell length and width were about (1 ~ 2) μm and 0.5 μm, respectively (Figure 1C). This bacterium was Gram-negative, and catalase, indole, nitrate reduction, oxidation and fermentation of glucose, starch hydrolysis and urease tests were all positive. Casein hydrolysis, citrate utilization, gelatin, hydrogen sulfide and phenylalanine deaminase tests were negative.

### Growth improvement of *Spodoptera litura* by *Raoultella terrigena* with diets containing *Polygonum viviparum* powder

**Figure 2:**
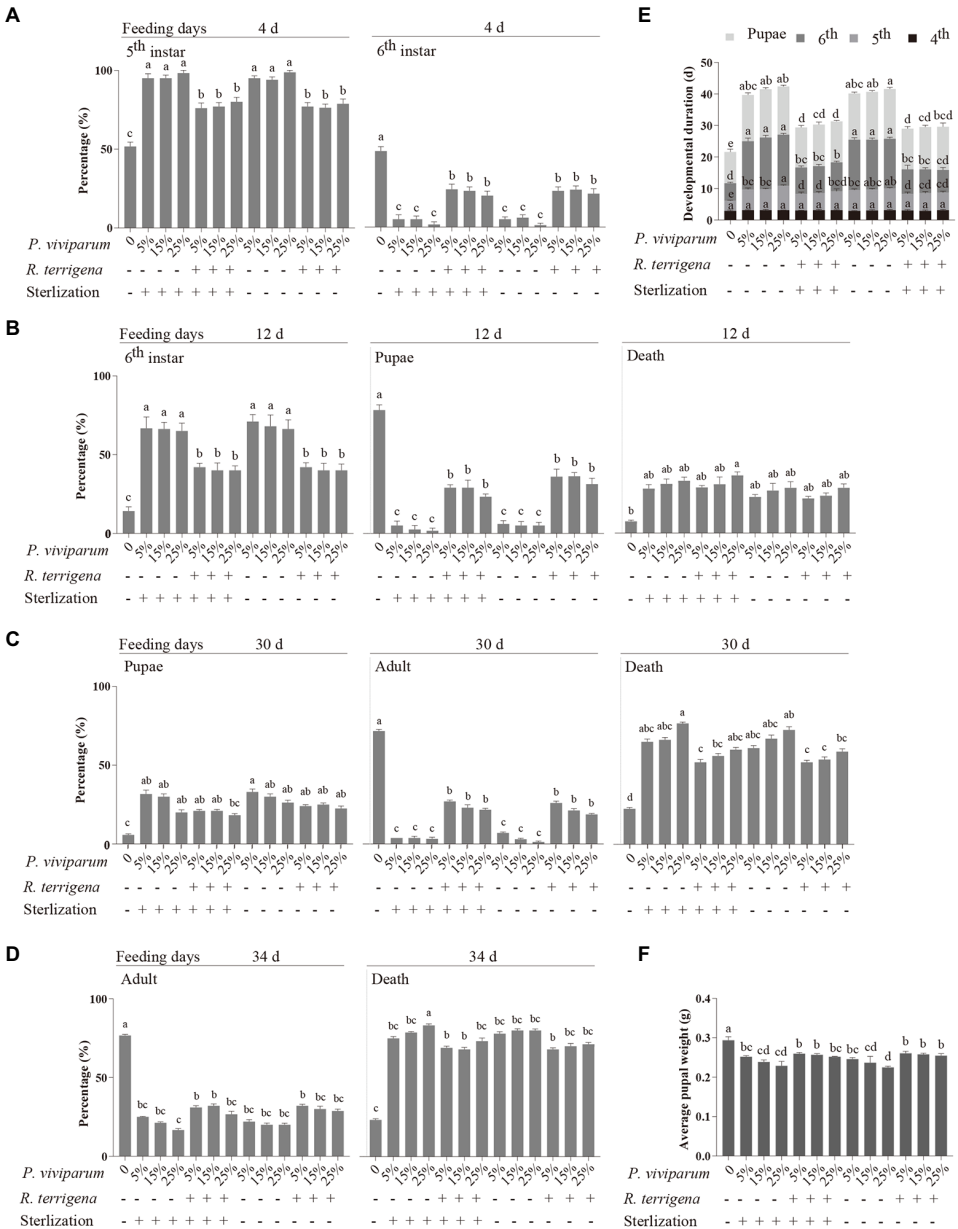
Development of the fourth instar larvae of *Spodoptera litura* reared on the artificial diets supplemented with 5, 15 or 25% *P. viviparum* root powder which was cocultured with or without *R. terrigena* for 7 days. Percentages of different developmental stages of *S. litura* for 4 day **(A),** 12 day **(B)**, 30 day **(C)** and 34 day **(D)**, developmental durations of the different developmental stages of *S. litura* **(E)** and average pupal weight of *S. litura* on the first day post pupation **(F)**.

*Polygonum viviparum* exhibits inhibitory activity for the growth of *S. litura* larvae (Qiu and Han, 2016), which was confirmed in this study. The growth of the 4th instar *S. litura* larvae fed with the artificial diet containing 5, 15 and 25% *P. viviparum* root powder was significantly hampered, compared with those fed with the artificial diet without *P. viviparum*. Surprisingly, the growth was significantly improved when the artificial diet contained *P. viviparum* root powder cocultured with *R. terrigena* (Figure 2). Notably, the percentages of the 6th instar larvae at day 4 (Figure 2A), pupae at day 12 (Figure 2B) and adults at day 30 (Figure 2C) in six treatments with *R. terrigena*-fermented *P. viviparum* root powder were all significantly higher than those without *R. terrigena*. The final adult emergence rate at day 34 in the control without *P. viviparum* in the artificial medium was significantly higher than those fed with the artificial diet containing *P. viviparum*, no matter whether *R. terrigena* was present or not (Figure 2D).

The developmental durations of the 4th, 5th, 6th instar larvae and pupae were further compared in details among the artificial media with or without *R. terrigena*. No significant difference was observed in the developmental duration of the 4th instar larvae fed with different diets. However, for the 5th, 6th instar larvae and pupae, the developmental durations in the treatments without *P. viviparum* root powder were 3.2, 5.6 and 9.9 days, respectively, which were significantly shorter than those in the treatments containing different concentrations of *P. viviparum* powder. The presence of autoclaved or unautoclaved *R. terrigena* cultures containing three concentrations of *P. viviparum* in the diets significantly shortened the developmental durations of the 5th, 6th instar larvae and pupae of *S. litura* (Figure 2E). Furthermore, the average pupal weight in the control without *P. viviparum* in the artificial medium was significantly higher (0.29 g) than those fed with the artificial diet containing *P. viviparum*, no matter whether *R. terrigena* was present or not and the average pupal weight was significantly higher in the artificial diet mixed with autoclaved *R. terrigena* culture containing 15% *P. viviparum* powder or in the artificial diets mixed with unautoclaved *R. terrigena* culture containing 15% or 25% *P. viviparum* powder, compared with that in the diets without *R. terrigena* coculture (Figure 2F).

In all, these results indicated that the growth of *S. litura* with diets containing *P. viviparum* powder was improved by the presence of *R. terrigena* bacteria.

### Decreased concentration of quercetin in *Polygonum viviparum* powder and in LB medium by *Raoultella terrigena*

**Figure 3:**
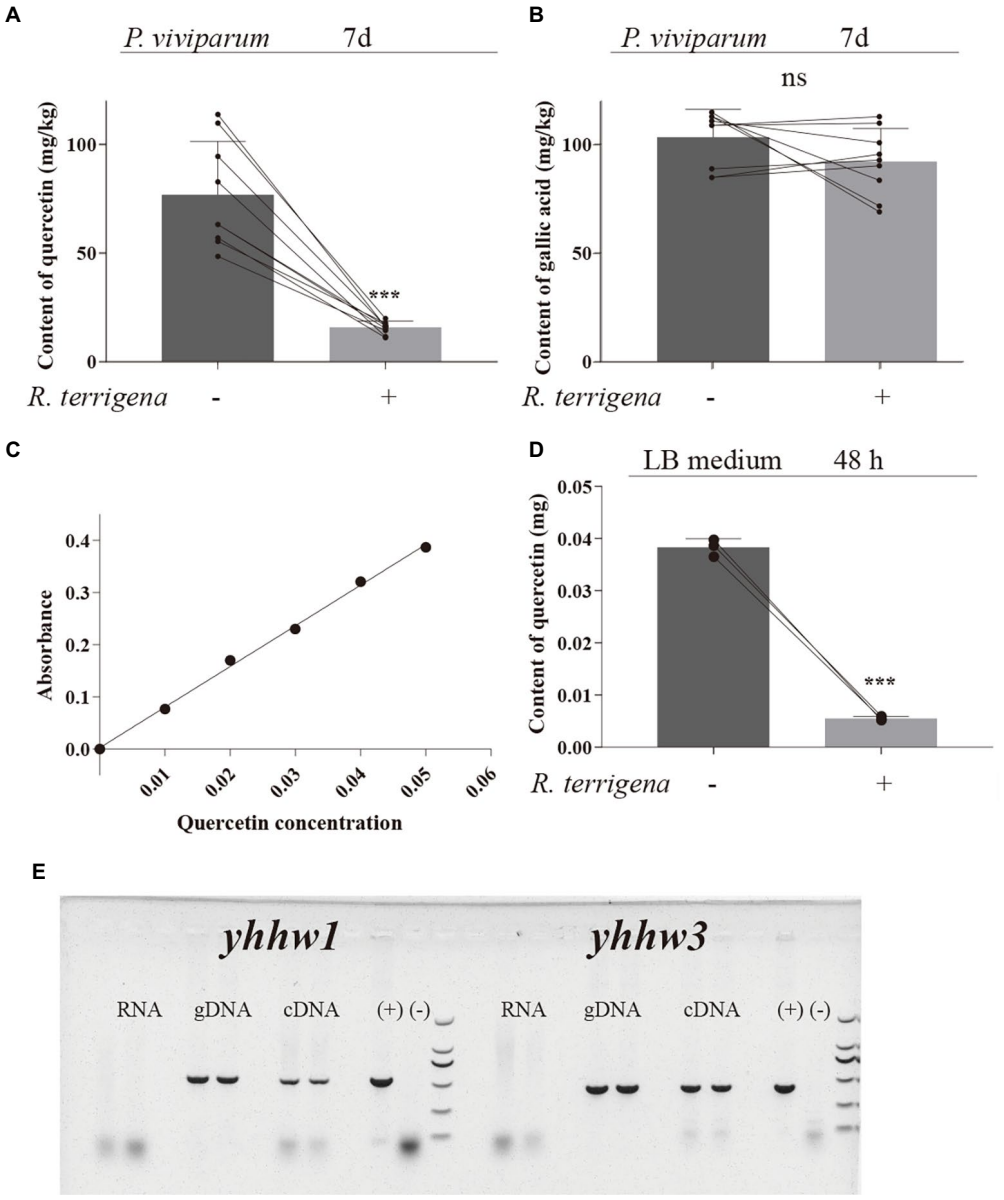
Quercetin degradation by *R. terrigena* in *P. viviparum* powder and in LB medium. Contents of quercetin **(A)** and gallic acid **(B)** analyzed by HPLC-DAD, in 25% *P. viviparum* root powder fermented with or without *R. terrigena* for 7 days, standard curve of quercetin added in the LB culture medium **(C)** and quercetin degradation in LB medium with or without *R. terrigena* cultured for 48 h **(D)**, expression of the genes *yynw1* and *yynw3* in natural larvae of *T. xiaojinensis* **(E)**. Electrophoresis of the products for the different templates used for RT-PCR of the genes *yynw1* and *yynw3*. No product was detected for the total RNA indicating the RNA without contamination of DNA. A band of ~500 bp for yynw1 and A band of ~400 bp for yynw3 were obtained for both cDNA and DNA. (+) positive control, DNA product from *R. terrigena*. (−) non-template control.

To investigate whether growth improvement of *S. litura* by *R. terrigena* with diets containing *P. viviparum* powder was caused by the reduced flavonoid content, the content of quercetin and gallic acid in *P. viviparum* powder with or without *R. terrigena* was analyzed by HPLC. The average concentration of quercetin in *P. viviparum* root powder was 76.70 mg/kg, but only 15.74 mg/kg in the culture with *R. terrigena* after 7 days, in which 79.48% quercetin were degraded (Figure 3A). Although the concentration of gallic acid was higher than that of quercetin in *P. viviparum* powder, no significant difference was observed in the content of gallic acid in *P. viviparum* powder between the cultures with or without *R. terrigena* (Figure 3B). These results showed that *R. terrigena* could degrade quercetin but not gallic acid in *P. viviparum* powder.

Decreased concentration of quercetin by *R. terrigena* was further demonstrated in LB medium containing quercetin only. The supernatants from the bacterial culture with quercetin were colorless, while LB medium with quercetin but without *R. terrigena* were yellow (Supplementary Figure S4). According to the quantitative test, a standard curve of quercetin was established. The linear regression equation of concentration logarithm was y = 7.794 × −0.0026 and the correlation co-efficiency R was 0.9975 (Figure 3C). The reduction rate of quercetin by *R. terrigena* reached 85.56% (Figure 3D). Together with the isolated strain B49, *R. terrigena* ATC700372 (GDMCC1.452) also exhibited the ability of degrading quercetin. The supernatants from the *R. terrigena* ATC700372 bacterial culture with quercetin were also colorless (Supplementary Figure S5) and its quercetin reduction rate reached 75.80% (Supplementary Table S1). The results further indicated that *R. terrigena* could degrade quercetin.

To further determine the contribution of *R. terrigena* to quercetin degradation in *T. xiaojinensis*, the genes *yynw1* and *yynw3* with quercetinase activity were screened in *R. terrigena* and the expression of these two genes was investigated in *T. xiaojinensis*. The obtained sequences of *yynw1* and *yynw3* were listed in Supplementary Table S2. The results showed that *R. terrigena* possessed the genes *yynw1* and *yynw3*. The expression of the genes *yynw1* and *yynw3* was also confirmed by using reverse transcriptase PCR from RNA extracts of field-collected larvae (Figure 3E), indicating a contribution of *R. terrigena* to the process of quercetin degradation in natural populations of *T. xiaojinensis*.

### Growth improvement of *Spodoptera litura* by *Raoultella terrigena* with diets containing quercetin

**Figure 4:**
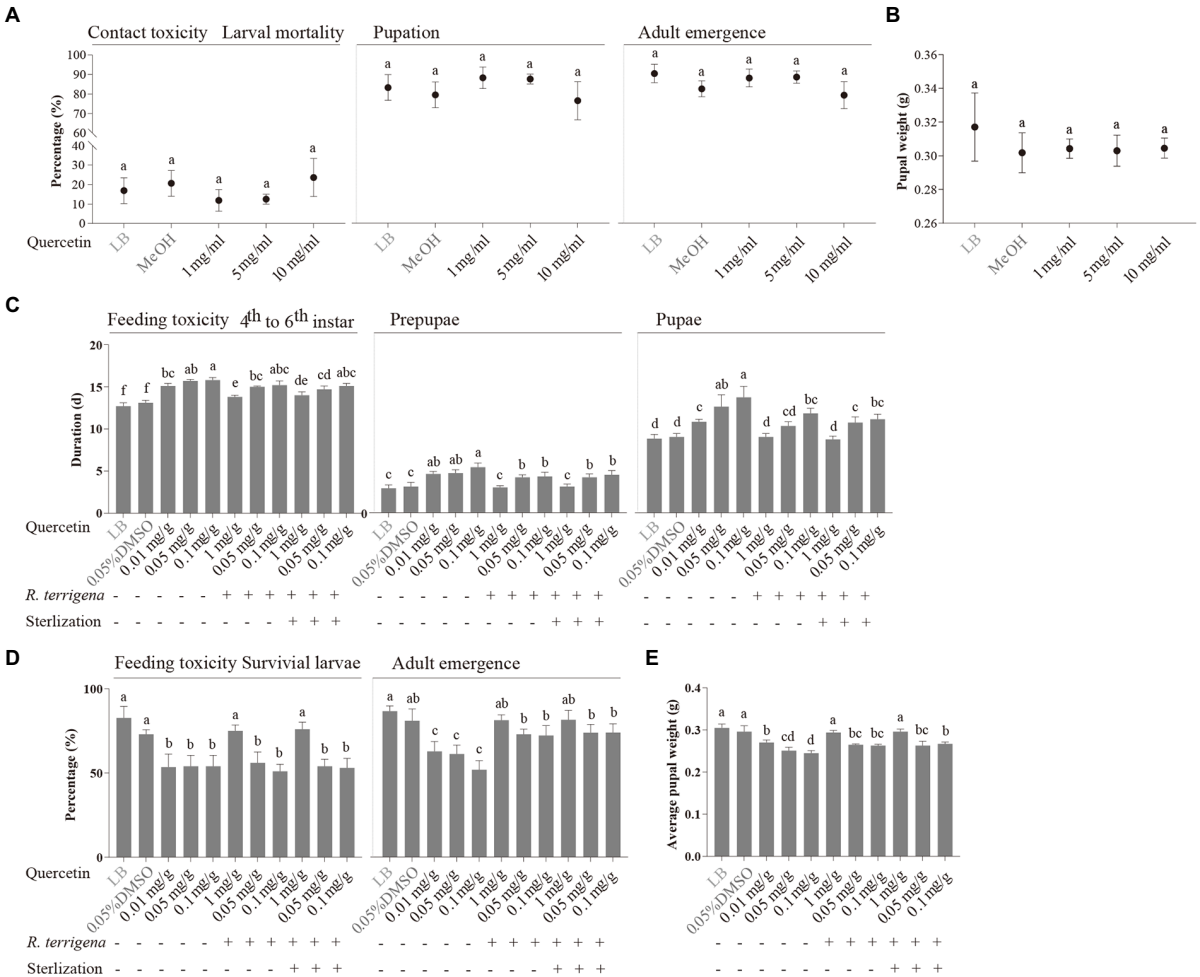
Toxicity of quercetin against *S. litura*. Effects of quercetin on larval mortality, pupation rate, adult emergence rate **(A)** and average pupal weight **(B)** of *S. litura* using filter paper diffusion assay, developmental durations of the different developmental stages of *S. litura* **(C)**, percentages of survival larvae and adult emergence **(D)**, and average pupal weight **(E)** of *S. litura* reared on the artificial diets containing 0.01, 0.05 or 0.1 mg/g quercetin which was cocultured with or without *R. terrigena* for 48 h.

Growth improvement of *S. litura* by *R. terrigena* was further confirmed in diets containing quercetin at three concentrations (1, 5 and 10 mg/ml). Quercetin at three concentrations showed no toxicity against *S. litura* in filter papers, as larval mortality, pupation rate, emergence rate (Figure 4A) and pupal weight (Figure 4B) of *S. litura* were not significantly different from those in the negative and methanol solvent control. However, the results from the feeding toxicity assay showed inhibitory effect of quercetin on the growth of *S. litura*. The 4th instar larvae fed with the artificial diets, respectively, with 0.01, 0.05 and 0.1 mg/g quercetin exhibited significantly longer developmental durations for later larvae, prepupa and pupa than those fed with the artificial diet without quercetin (Figure 4C). Furthermore, the larval survival rate, adult emergence rate (Figure 4D) and pupal weight (Figure 4E) significantly decreased in the diets containing quercetin at three concentrations.

When the artificial diet was mixed with quercetin at 0.1 mg/g degraded by *R. terrigena* and used to feed on *S. litura* 4th instar larvae, insect growth was improved, including shortened larval, prepupal, pupal durations and increased larval survival rate, adult emergence rate as well as average pupal weight, no matter if the cultures of quercetin and *R. terrigena* were autoclaved or not (Figure 4). Likewise, larval, prepupal and pupal durations were significantly shortened with quercetin at both 0.05 mg/g and 0.1 mg/g (Figure 4C). Both adult emergence rate (Figure 4D) and average pupal weight (Figure 4E) were significantly increased although larval survival rate was not improved when *S. litura* 4th instar larvae were fed with the diets containing quercetin at 0.05 and 0.1 mg/g.

Taken together, these results further confirmed that quercetin in *P. viviparum* root powder was involved in the growth hamper of *S. litura* and co-culture of quercetin and gut bacterium *R. terrigena* could relieve the growth hamper.

## Discussion

Herbivorous insect microbiota has been shown to mediate the outcome of insect-plant interaction in different ways, such as essential nutrient supplementation, xenobiotic compounds degradation and plant defense suppression (Douglas, 2009; Kohl and Dearing, 2012; Cheng et al., 2017; Shukla and Beran, 2020). Microbiota of some herbivorous insects have been demonstrated to contribute to the detoxification of plant secondary metabolites (Kohl et al., 2014; Ceja-Navarro et al., 2015; Berasategui et al., 2017), however, bacterial involvement of quercetin degradation in plants as insect food remain unclear. This study demonstrated that isolated gut bacterium *R. terrigena* from *T. xiaojinensis* larvae are involved in the degradation of quercetin in *P. viviparum* root powder to relieve the growth suppression of herbivorous insects such as *S. litura* and *T. xiaojinensis*.

Nine bacterial species were isolated from the guts of field species of *T. xiaojinensis* on mineral medium containing 10% *P. viviparum* as the sole source of carbon and nitrogen, but only *R. terrigena* could normally multiply in liquid mineral medium containing 25% *P. viviparum*. *R. terrigena* was also detected from the sterilized bud and root tissues of *P. viviparum*, indicating *R. terrigena* as a gut bacterium from *T. xiaojinensis* larvae and an endophyte bacterium in *P. viviparum*.

Microbial communities from *Thitarodes* larvae have been investigated using traditional culture-dependent and culture-independent methods. Eight bacterial genera (*Staphylococcus*, *Bacillus*, *Klebsiella*, *Pseudomonas*, *Aeromonas*, *Plesiomonas*, *Sporosarcina* and *Neisseria*) are isolated from the guts of wild *T. gonggaensis* larvae (Zhuo et al., 2004). Eight bacterial genera (*Enterobacter*, *Carnobacterium*, *Novosphingobium*, *Acinetobacter*, *Pseudomonas*, *Klebsiella*, *Pantoea* and *Delftia* are also obtained from the guts of wild *T. gonggaensis* larvae (Liu et al., 2008). Several fungal genera (*Mortierella*, *Trichosporon*, *Mucor*, *Rhinocladiella*, *Cephalosporium*, *Rhodiola* and *Mastigobasidium*) are identified by RFLP analysis from this ghost moth species, and *Cryptococcus magnus*, *Geomyces* sp. and *Trichosporon porosum* by culture method (Yu et al., 2008). The microbiota of the hemolymph and gut from *T. xiaojinensis* larvae with or without injected *O. sinensis* blastospores are investigated by culture-dependent and-independent methods, and 25 culturable bacterial species and 14 fungal species, together with 537 bacterial OTUs and 218 fungal OTUs, are identified from the hemolymph and gut of samples from five infection stages (Wu et al., 2020). However, *R. terrigena* has not been obtained from all these reports. This may reflect the differences in sampling locations, isolation methods and larval culture methods. *R. terrigena* isolated from the three wild-collected *Thitarodes* population but not from the three corresponding artificial rearing population further indicated the difference of larval gut microbiota caused by sample source (Liu et al., 2021).

Endophytes as a ubiquitous associate of the plant are considered to play a pivotal role in regulating the primary and secondary metabolism of their host plant, and in providing elicitors to improve plant health, ameliorating stress tolerance in plants and source of therapeutically important secondary metabolites (Pandey et al., 2022). *R. terrigena* is considered to be an environmental bacterium, which is isolated mainly from soil and aquatic environments (Fazal et al., 2019). It is also isolated from plant roots of *Nicotiana tabacum* and switchgrass *Panicum virgatum*, and considered to be an endophytic nitrogen-fixing bacterium (Schicklberger et al., 2015; Gushgari-Doyle et al., 2021). Furthermore, *R. terrigena* is detected from the gut of *Dendroctonus rhizophagus* and *D. valens*, exhibiting high acetylene reduction activity (Morales-Jimenez et al., 2013), as well as from the reproductive system of the female oriental fruit fly *Bactrocera dorsalis*, and its metabolites are found to be attractive to adults in field trapping assays (Shi et al., 2012). Surprisingly, in this study, *R. terrigena* was firstly verified to effectively degrade quercetin in *P. viviparum* roots, which are natural food for *Thitarodes* larvae, and to improve *S. litura* growth when the larval medium contained *P. viviparum* root powder or quercetin. Because no artificial medium is available for *Thitarodes* larvae, whether the quercetin carried by *P. viviparum* roots is toxic to *Thitarodes* larvae is not validated. Why this endophyte bacterium from *P. viviparum* metabolizes quercetin inside the plant for the improved herbivorous insect growth needs further exploration.

Quercetin is considered to be a kind of flavonoid widely present in terrestrial plants and herbal medicines which helps plants resist herbivore attack (Simmonds, 2003; Selin-Rani et al., 2016; Shi et al., 2021), and induces expressions of some detoxification enzymes in insects (Zhang et al., 2012; Shi et al., 2021). Quercetin is assumed to be an ecofriendly alternative compound not only for *S. litura* but also for *Lymantria dispar*, *Helicoverpa zea*, *Oedaleus asiaticus*, *Aphis* spp. and *Callosobruchus chinensis* (Lattanzio et al., 2000; Salunke et al., 2005; Selin-Rani et al., 2016; Cui et al., 2019). The synergistic effect of quercetin and nucleopolyhedrovirus was also found (Shi et al., 2021). However, quercetin metabolism by microbes is still unclear (Aboshi et al., 2014). In this study, quercetin degradation by *R. terrigena* was discovered, which provides new techniques for mass production of *Thitarodes* larvae by employing this bacterium onto *P. viviparum* roots and seeds, and for the control of insect pest by inhibiting this gut bacterium when the pests feed on the plants full of quercetin.

## Conclusion

*Polygonum viviparum* roots are toxic to *S. litura*, but favorable food for *T. xiaojinensis* larvae in the Himalaya Mountain. *R. terrigena* was firstly isolated from the gut of *T. xiaojinensis* larvae and *P. viviparum* roots and seeds. This bacterium grew in liquid mineral medium containing 25% *P. viviparum* root powder. The artificial medium containing 5, 15 and 25% *P. viviparum* powder showed significant growth inhibition of *S. litura*, but the growth was significantly improved when *R. terrigena* was present. Quercetin in *P. viviparum* powder was found to involve in the suppressed growth of *S. litura*. Quercetin degradation by *R. terrigena* was demonstrated to reduce the growth suppression of *S. litura*. These results provide insight into the detoxification of gut microbes from insects in the interaction between herbivores and defensed host plants.

## Data availability statement

The datasets presented in this study can be found in online repositories. The names of the repository/repositories and accession number(s) can be found at: GenBank accession number MW555198.1, https://www.ncbi.nlm.nih.gov/nuccore/MW555198.1/.

## Author contributions

GL and RH designed the research and wrote or revised the manuscript. GL and LC conducted the research. All authors contributed to the article and approved the submitted version.

## Funding

This study was supported by Guangdong Basic and Applied Basic Research Foundation (2021A1515010520), Guangzhou Municipal Science and Technology Bureau (202102080238), Qinghai Science and Technology Major Project (2021-SF-A4-1) and GDAS Special Project of Science and Technology Development (2020GDASYL-20200103097 and 2022GDASZH-2022010106).

## Conflict of interest

The authors declare that the research was conducted in the absence of any commercial or financial relationships that could be construed as a potential conflict of interest.

## Publisher’s note

All claims expressed in this article are solely those of the authors and do not necessarily represent those of their affiliated organizations, or those of the publisher, the editors and the reviewers. Any product that may be evaluated in this article, or claim that may be made by its manufacturer, is not guaranteed or endorsed by the publisher.

## Footnotes

^1^ http://www.ncbi.nlm.nih.gov/
