# Supplementary material for: Plant quercetin degradation by gut bacterium Raoultella terrigena of ghost moth Thitarodes xiaojinensis
Source: Front Microbiol. 2022 Dec 22;13:1079550. doi: 10.3389/fmicb.2022.1079550 (PMC9815537; doi:10.3389/fmicb.2022.1079550)
Supplement: Supplementary file 1 [file Data_Sheet_1.docx]

Supplementary Material

## Supplementary Figures


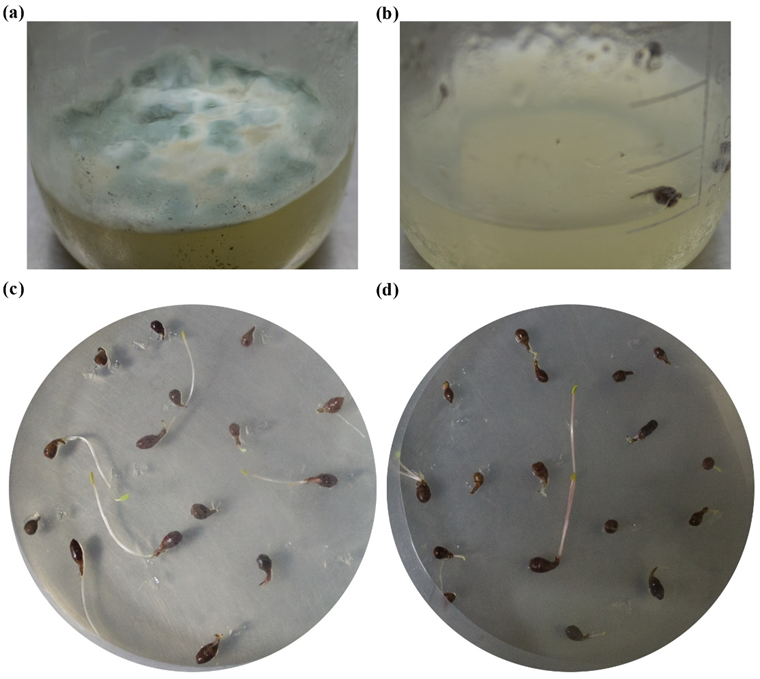


**Supplementary Figure 1.** Determination of the effectiveness of surface sterilization of *Polygonum viviparum* seeds. A and B, surface-unsterilized and -sterilized seeds cultured on LB medium; C-D, buds and root of surface-sterilized seeds cultured on mineral media.

**
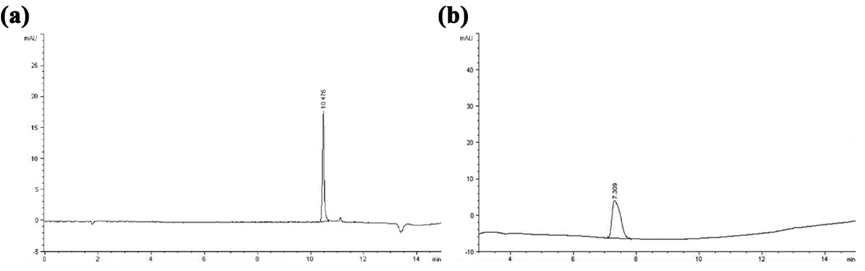
**

**Supplementary Figure 2.** HPLC chromatograms for the determination of quercetin (A) and gallic acid (B).


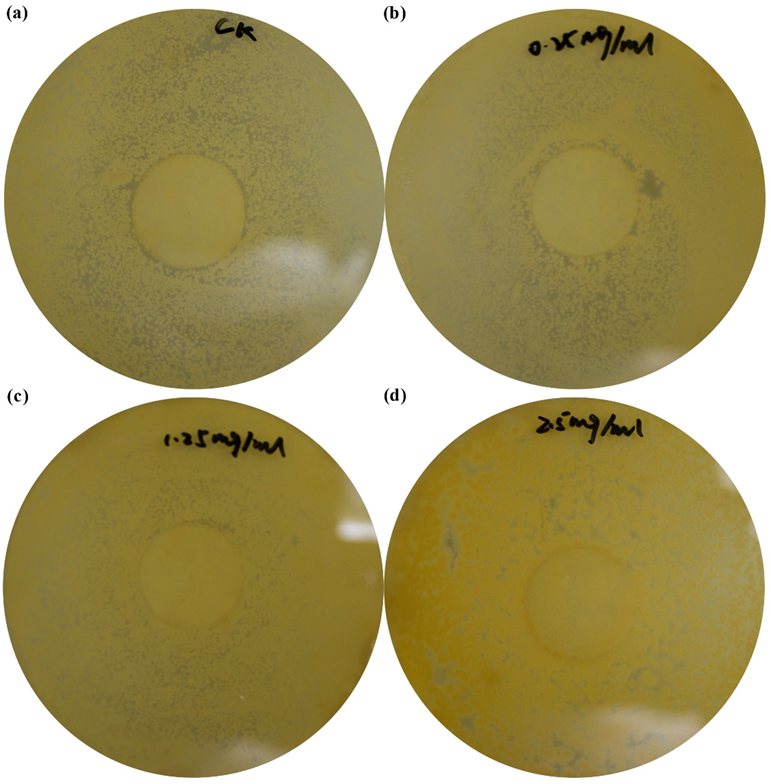


**Supplementary Figure 3.** Effect of different concentrations of quercetin on the growth of *Raoultella terrigena* tested by filter paper method. A, control, sterilized water; B, 0.25 mg/mL quercetin; C, 1.25 mg/mL quercetin; D, 2.5 mg/mL quercetin.


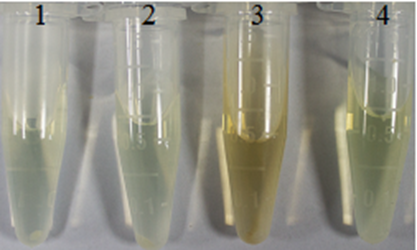


**Supplementary Figure 4.** *In vitro* quercetin fermentation supernatants by *R. terrigena* B49 cultured for 48 hours. tube 1 was a negative control without *R. terrigena* and quercetin inoculation, tubes 2 and 4 were inoculated with *R. terrigena* and a combination of *R. terrigena* and quercetin, respectively, tube 3 was a positive control inoculated with quercetin only.


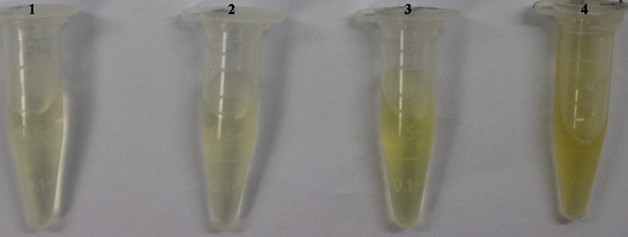


**Supplementary Figure 5.** *In vitro* quercetin fermentation supernatants by *R. terrigena* ATC700372 (GDMCC 1.452) cultured for 48 hours. tube 1 was a negative control without *R. terrigena* and quercetin inoculation, tubes 2 and 3 were inoculated with *R. terrigena* and a combination of *R. terrigena* and quercetin, respectively, tube 4 was a positive control inoculated with quercetin only.

## Supplementary Tables

**Supplementary Table 1.** Quercetin degradation in LB medium by *R. terrigena* strain ATC700372 cultured for 48 hours.

| **Treatment** | **Absorbance** | **Quercetin in 1 mL LB (mg)** | **Degradation rate（%）** | **Average degradation rate (±SD)（%）** |
| --- | --- | --- | --- | --- |
| ATC700372 | 0.063 | 0.0078 | 80.52 | 75.80±4.24 |
|  | 0.075 | 0.0098 | 74.57 |  |
|  | 0.086 | 0.0108 | 72.32 |  |
| Control | 0.313 | 0.0398 |  |  |
|  | 0.287 | 0.0365 |  |  |
|  | 0.304 | 0.0387 |  |  |

**Supplementary Table 2.** Sequences of the two genes *yhhw1* and *yhhw3* in *R. terrigena*.

| **Gene name** | **Sequences (5’-3’)** |
| --- | --- |
| *yhhw1* | TGGCTCGATTCCTGGCATACTTTCTCTTTCGCCAACTATTACGACGCCAATTTTATGGGCTTTTCCGCTCTGCGCGTGATTAACGATGACGTGATTGACGCCGGCCAGGGCTTTGGGACCCACCCGCATAAAGATATGGAAATTCTGACCTACGTGCTGGAAGGGGTGGTTGAACACCAGGACAGCATGGGTAACAAAGAGCAGGTTCCTGCGGGTGAATTCCAGATCATGAGCGCCGGTACCGGGGTTCGTCACTCTGAATACAACCCGAGCAGCACCGAGCGTCTGCACCTGTATCAAATCTGGATTATGCCGGAAGAGAACGGTATCGCTCCGCGCTATGAGCAGCGCCGGTTTGATGCGCTGCAGGGCAAACAGCTGGTGCTGTCGCCGGACGCTCGCGAAGGCTCGCTGAAAGTGCATCAGGATATGGAACTGTACCGCTGGGCGCTGCTGAAAGATGAGCAGTCGGTCCATCAGATTGCCGCGGAACGTCGCGTGTGGATTCAGGTCGTCA |
| *yhhw3* | TGCCGGCCAAAGACAAAATGGCCGCGCCCGGCTACCAGAGCATCACTGCTGACCTCATTCCCCGCGTCGCGCTGCCGGATAACGCAGGCCACGTTCGGGTCATCGCCGGTGATTACGCCGACGTTTCCGGCCCGGCGCGCACCTTCTCGCCGCTGAACGTCTGGGATATGCAGCTGACGCAGGGCCACGACGTCTCGTTACGCCAGCCTGCCGGCTGGAGCACCGCGCTGGTTGTTCTTGAAGGTGAAATCAGCGTTAACGGCGCTGAAAATGCGCGCGAAGGGCAGCTGGTGGTGTTGAGTCAGCAGGGTGAAACGCTGCACCTGAGCGCTACCGCTGATGCCAAAATCCTGCTGCTGGCCGGCGAACCGCTGGGTGAGCCTATTGTCGGCTATGGCCCATTT |
